# Examining the consumer view of refreshing perception, relevant fruits, vegetables, soft drinks, and beers, and consumer age and gender segmentations

**DOI:** 10.1002/fsn3.2857

**Published:** 2022-03-29

**Authors:** Jessica L. Ramirez, Amy Hampton, Xiaofen Du

**Affiliations:** ^1^ Department of Nutrition and Food Sciences Texas Woman’s University Denton Texas USA

**Keywords:** consumer segment, eating and drinking behavior, emotion in food, refreshed, refreshing, refreshment

## Abstract

Consumer perspective of refreshing perception is underexplored, despite it being an emotional attribute to describe foods, beverages, hygiene products, and household items. An online survey (*N* = 1518) was designed to collect consumer insight into the importance of refreshing, the definition and factors related to it, and the identification of refreshing fruits, vegetables, and drinks. Nearly all participants (99.8%) cited that they have had the need to consume a food or beverage to feel refreshed, and 76.3% cited that they need this at least once per day. The factors most associated with refreshing were thirst‐quenching (84.1%), temperature (86.2%), and cooling taste (86.0%). Water (86.6%), watermelons (80.8%), and cucumbers (83.5%) were the beverages/foods most frequently specified as refreshing. A second survey (*N* = 1050) examined refreshing perception specifically related to beer consumption and associated flavor. Beer was rated highly refreshing by 75.5% of participants, affirming its refreshing reputation. Refreshing perceived from beer was most associated with cool temperature (95.4%), flavor of the beer (88.6%), lightened mood (87.1%), and thirst‐quenching (49.0%). Beers with crisp/clean flavors (87.3%) and citrus flavors (35.7%–51.7%) were most frequently specified as refreshing. There were no gender differences in the definition of refreshing and associated thirst‐quenching and cold, although age differences in defining beer refreshing were significant (*p* ≤ .05). There were significant gender and age differences in types of refreshing vegetables, soft drinks, beer flavors, and varieties. The study provided consumer insight into refreshing perception and the gained knowledge could be used in new product design.

## Graphical abstract

This study demonstrated the importance of refreshing perception for consumer, the definition and factors related to the refreshing, types of fruits, vegetables, soft drinks, and beers perceived refreshing, as well as consumer segments based on age and gender.

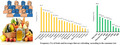

## 1 INTRODUCTION

Since the 1980s, research related to food‐evoked emotion has gained increasing attention in the areas of marketing and advertising, in which emotion is used as a critical tool to predict consumer food choice (Ng & Hort, 2015). Marketing researchers use the emotional response data to influence consumer food purchase intent, brand choice, purchase decision, and consumption habit, while sensory scientists employ emotion research in new food product development to elicit positive emotional response, consequently influencing consumer choice behavior (Ng & Hort, 2015). Currently, food product associated emotional quality is becoming increasingly important for differentiation in highly competitive and mature markets, particularly when products within the same category have a high variety from which to be chosen, and are similar in quality and price (Ng & Hort, 2015; Schifferstein et al., 2013).

The term refreshing, including related words such as refreshment, refreshed, and occasionally freshness, is considered an emotional response to the food consumption experience and is often used to characterize certain types of foods and beverages. For example, refreshing is one of the positive or pleasant emotional terms used for products such as wine, beer, beverages, and salad, when multiple emotional responses are included in these studies (Chaya et al., 2015; Chonpracha et al., 2020; Geier et al., 2016; Mora et al., 2020; Ramirez et al., 2020). The majority of these studies were published in the recent decade, indicating that the refreshing concept is an important aspect of the food consumption experience and is gaining increasing attention. The understanding of an emotion‐related response of refreshing, however, is still very limited.

Refreshing is generally one of the terms in the consumer‐generated emotion lists. The Geneva Emotion and Odor Scale (GEOS) questionnaire is developed with adjectives for emotions and olfaction, which results in 36 terms and are divided into six dimensions (Chrea et al., 2009; Delplanque et al., 2012; Ferdenzi, Delplanque, et al., 2013; Ferdenzi, Roberts, et al., 2013; Ferdenzi et al., 2011; Porcherot et al., 2010). Refreshing is one of the dimensions, including three terms “energetic,” “clean,” and “refreshed.” The emotion lexicon for EsSense Profile consists of 39 terms, which are categorized as “positive,” “negative,” and “unclassified” groups (King & Meiselman, 2010). “Energetic” is one of the positive emotions including refreshment, which is consistent with GEOS terms. EsSense Profile is further confirmed by a consumer‐defined lexicon–CATA (check‐all‐that‐apply) approach (CD–CATA) and indicates “refreshed” is one of the positive terms that consumers selected (Ng et al., 2013a, 2013b). A study classifying feelings led to 23 clusters of positive effects, and “refreshed” belongs to one of the clusters and includes energetic and vigorous in the same cluster (Thomson & Crocker, 2013). Additionally, a food‐associated emotion lexicon is designed to understand the role that emotions play in food consumption experiences (Gmuer et al., 2015). “Refreshing” is one of the most frequently used emotion or feeling lexicons, selected by 98.2% of the participants. These studies indicate that refreshing is an important emotion term that consumers can perceive and select to describe their positive feelings.

A few studies have investigated the definition of refreshing. Refreshing has been defined as “a way to restore strength and animation, revive, arouse, stimulate, and contain thirst‐quenching properties” (Labbe et al., 2009). Several studies involving the refreshing concept have emphasized its key role in the thirst‐quenching and cooling properties of foods and beverages (Guinard et al., 1998; McEwan & Colwill, 1996; van Belzen et al., 2017). These studies indicate that refreshing is a multidimensional concept that is based on consumer opinions and specified food categories.

The major driver for refreshing perception is most likely the sensory qualities of a food, though the relationship between sensory characteristics and emotions remains underexplored (Spinelli & Jaeger, 2019). It has been suggested that the temperature, flavor, and texture of foods have an impact on how refreshing are foods perceived to be, when asked to list sensory characteristics of refreshing foods and beverages (Roque et al., 2017, 2018; Zellner & Durlach, 2002). Positive correlations between refreshing and acidity, astringency, fruity flavor, and strength of flavor in assorted beverages are reported, while a negative correlation is found between refreshing and sweetness (McEwan & Colwill, 1996). The findings that acidity and sweetness are positive and negative drivers of refreshing, respectively, are confirmed by a study of flavored gels (Labbe et al., 2009). In beers, however, acidity, bitter, malty, hoppy, burnt, and metallic notes are all negatively associated with the refreshment (Guinard et al., 1998). Beers with more flavors are less refreshing, while popsicles with more flavors are positively correlated with refreshing. All flavored popsicles are more refreshing than their unflavored counterparts, while lemon and mint flavored popsicles are particularly more refreshing than raspberry (van Belzen et al., 2017). Sweet taste, however, was not associated with refreshing (van Belzen et al., 2017). The inconsistent role of flavor (aroma and taste) in the refreshing perception found across those studies suggests that there is room for further investigation.

This study aimed to examine consumer perspective on the importance of refreshing, the definition and factors related to the refreshing perception, and related refreshing fruits, vegetables, and drinks in general, as well as consumer segments of age and gender. Our previous sensory studies indicate that watermelon flesh is refreshing for its flavor, texture, and temperature (Ramirez et al., 2020, 2021). Watermelon is included in the current study to further verify the concept of refreshing.

## 2 MATERIALS AND METHODS

### 2.1 Participants

All consumer study procedures were reviewed and approved by the Texas Woman's University (TWU) Institutional Review Board (IRB). Survey participants were recruited using a TWU email list with a pool of approximately 18,000 people to advertise and deliver the questionnaire to university students, faculty, and staff at three campuses (Denton, Houston, and Dallas, TX, USA). The only discriminating factor was the consent age of 18 for the survey related to the general refreshing concept and the age of 21 for the survey related to beer. The surveys were administered on the Internet using Google Forms and were designed to be completed within 10–15 min, though participants were instructed to take as much time as needed. Participants were compensated with a chance to win a gift card after completion of the survey.

### 2.2 Survey design

The consumer survey of the general concept of refreshing included eight questions, and the question types were five CATA and three single choice (SC) questions (Table 1A). These eight questions were designed to examine how important is refreshing perception, how it was defined and factors that influenced it, and fruits, vegetables, and beverages that were refreshing (Table 1A). Introductory survey questions addressed how often participants felt the need to consume something refreshing and the importance of a refreshing perception when deciding which food or beverage to consume. Then, participants were questioned about terms that described their definition of refreshing. Finally, consumers were asked to choose those that they felt were refreshing, given lists of 19 fruits, 14 vegetables, and 9 beverages. The lists were selected based on our preliminary survey and literature (Ferdenzi, Delplanque, et al., 2013). Demographic information was collected including participant gender, age, and education levels.

**TABLE 1:** Survey questions and question types for general survey (A) and beer survey (B)

	Question type	Question	Results
1A			
Significance	SC	How often do you feel the need to consume something refreshing?	Figure 1
	SC	When choosing something to DRINK, how important is it for the beverage to be refreshing?	Figure 2a
	SC	When choosing something to EAT, how important is it for the food to be refreshing?	Figure 2b
Definition	CATA	Which of the following describes your perception of refreshing?	Figure 5a
	CATA	Which of the following factors do you consider when deciding if a food or beverage is refreshing?	Figure 5b
Foods and drinks	CATA	Which of the following fruits would you describe as refreshing?	Figure 7a
	CATA	Which of the following vegetables would you describe as refreshing?	Figure 7b
	CATA	Which of the following beverages do you find refreshing?	Figure 7c
1B			
Significance	SC	How would you rate the refreshment of beer on a scale from 1 to 10 (1 is a very low refreshing perception and 10 is a very refreshing perception)?	Figure 3
	CATA	Why do you drink beer?	Figure 4a
	CATA	Which of these factors do you consider when you choose a beer to drink?	Figure 4b
Definition	CATA	When perceiving refreshment from beer consumption, do you feel…	Figure 6a
	CATA	Which beer factors impact your perception of beer refreshment?	Figure 6b
Beers	CATA	What type(s) of FLAVOR PROFILES do you find refreshing?	Figure 8a
	CATA	What type(s) of beer FLAVOR do you find refreshing?	Figure 8b
	SC	Do different seasons make a difference in which beer you find refreshing?	Figure 9
	CATA	What type(s) of beer do you find the most refreshing? (Answer set 1)	Figure 10
	CATA	What type(s) of beer do you find the most refreshing? (Answer set 2)	Figure 10

Note

Figures had corresponding results for each question.

Abbreviations: CATA, check‐all‐that apply; SC, single choice.

The beer‐focused survey was also designed to examine the significance of beer refreshing perception, how it was defined and factors that influence refreshment perceived from beer consumption, and specific beer flavor profiles/types that consumers most associated with the refreshing perception (Table 1B). The survey included eight CATA and two SC questions (Table 1B). A SC question asked participants to rate the refreshment of beer on a 1–10 intensity scale. The answer sets for the CATA questions that asked participants to select refreshing beer varieties were supplemented with examples of brands local to or accessible in Texas, which was where the survey was conducted. Demographic information was collected including participant gender, age, and education.

### 2.3 Statistical analysis

The frequencies of answers from the survey were obtained using Google Forms. Gender differences in survey responses for factors associated with refreshment and refreshing foods and beverages were analyzed by chi‐square tests using SPSS version 25 (IBM). Age differences in survey responses were explored by correspondence analysis (CA) using XLSTAT (Addinsoft). The CA plots were used to visualize the association between age and beer characters. The CA method could be referred to the literature (Costa et al., 2013; Kienstra & van der Heijden, 2015; Lana et al., 2017; Sharma et al., 2019). All statistical tests assumed a significance of *α* ≤ .05.

## 3 RESULTS AND DISCUSSION

### 3.1 Demographic information and data validation

There were a total of 1518 responses collected from the survey regarding the general concept of refreshing. Frequencies and percentages for the categorical demographic variables are displayed in Table 2A. The majority of participants were female (90.8%, *N* = 1369), aged 18–25 (48.6%), with at least some college level education. Despite the limitation of possible gender bias, the male population (*N* = 139) was considered high for a consumer study and effective for statistical analysis. The survey results were found to be consistent with the literature on refreshing as addressed in the discussion part of this paper and were considered valid.

**TABLE 2:** Frequencies and percentages for demographic variables of general survey with *N* = 1518 (A) and beer survey with *N* = 1050 (B)

	*n*	%		*n*	%
2A					
Gender			Education		
Male	139	9.21	Less than high school	3	0.2
Female	1369	90.79	High school	57	3.8
Age			Some college	424	27.9
18–25	737	48.6	Associate degree	189	12.5
26–35	390	25.7	Bachelor's degree	446	29.4
36–50	240	15.8	Master's degree	265	17.5
51–65	141	9.3	Doctorate	134	8.8
65 or older	10	0.7			
2B					
Gender			Education		
Male	262	25	High school	14	1.3
Female	782	74.5	Some college	179	17
Other	6	0.6	Associate degree	141	13.4
Age			Bachelor's degree	449	42.8
21–25	437	41.6	Master's degree	184	17.5
26–35	342	32.6	Doctorate	73	7
36–50	182	17.3	Other	10	1
51–65	76	7.2			
65 or older	13	1.3			

Note

*n* = frequency of participants; % = percent frequency.

The survey examining refreshment perceived from beer consumption obtained responses from 1050 participants whose demographic information is presented in Table 2B. Participants were mostly female (74.5%, *N* = 782) in the age range of 21–35 (74.2%) with college level education. Gender bias may have again been a limitation in this study, though the number of males (*N* = 262) was high for a consumer study and effective for statistical analysis. The results from this study were in line with findings that beer elicits a refreshed response (Guinard et al., 1998; Worch et al., 2020).

### 3.2 Significance of refreshing perception

**FIGURE 1:**
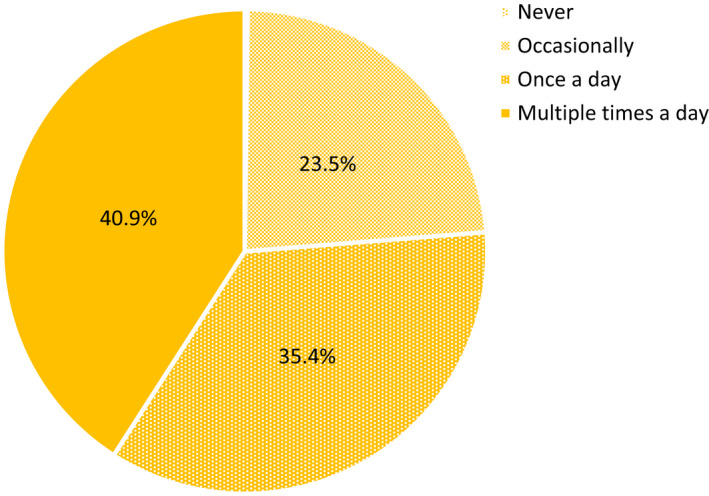
Frequencies (%) that consumers (*N* = 1518) felt the need to consume refreshing foods or beverages

**FIGURE 2:**
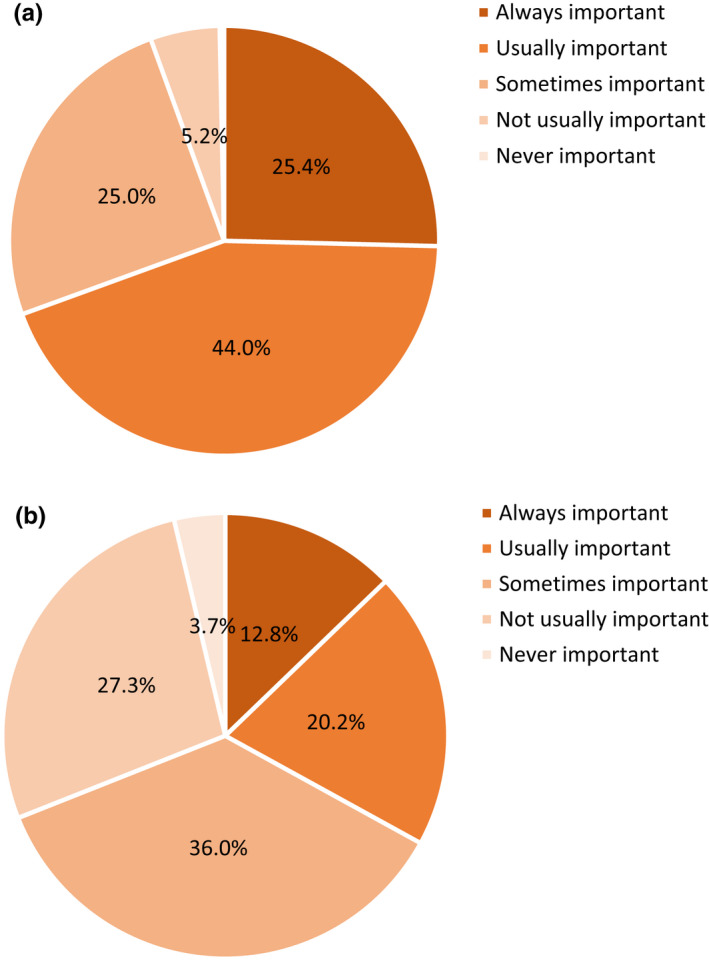
Frequencies (%) that consumers (*N* = 1518) felt important to have beverages (a) and foods (b) to be refreshing

The first survey showed the desire to consume a refreshing food or beverage at least once per day was reported by 76.3% of participants, while 23.5% needed to consume something refreshing occasionally (Figure 1). Less than 1% of participants reported that refreshing is something they never consider in their daily lives. More participants reported that refreshing was sometimes, usually, or always important for beverages compared to foods, 94.4% versus 69.0% (Figure 2). The results indicated that refreshing was found to be a daily requirement for the majority of consumers, suggesting that it fulfills a human need. This was unsurprising, given its correlation with the alleviation of thirst using beverages (Guinard et al., 1998; van Belzen et al., 2017; Van Zyl, 2016).

**FIGURE 3:**
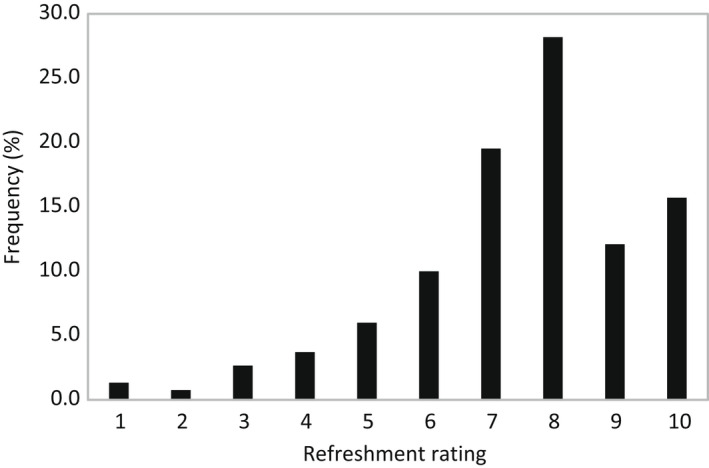
Frequency (%) that consumer (*N* = 1050) perceived refreshment of beer on a 1–10 category scale. *Y*‐axis number: 1 = very low refreshment; 10 = very high refreshment

**FIGURE 4:**
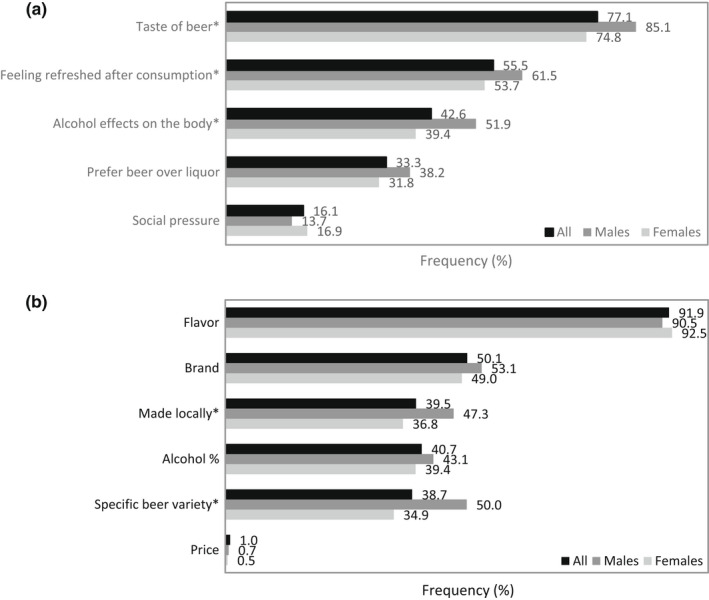
Percent (%) for the major reasons for consumer (*N* = 1050) drinking beer (a) and factors that affected choice of beer (b). *Chi‐square tests between genders with significance of *p* ≤ .05

The second survey indicated that the perceived refreshing from beer was rated at least a 7 on a 10‐point scale by 75.5% of survey participants (Figure 3). Within the reasons for drinking beer (Figure 4a), participants most frequently cited that they drank beer to enjoy its taste (77.1%), to feel refreshed (55.5%), and to experience the effect of alcohol on their body (42.6%). The importance of beer taste/flavor was further verified (Figure 4b). When asked about factors that affected their choice of beer, 91.9% of participants responded that they considered the flavor of beer to be important. About half of the participants expressed brand (50.1%), locality (39.5%), alcohol percentage (40.7%), and variety (38.7%) were important. The results indicated that taste or flavor of beer was the most impacted factor for beer drinking, while refreshing perception was an especially desired outcome during consumption of beer, which was consistent with literature (Ortal & Edahiro, 2020).

Despite the human need and desire for the refreshing sensation, few studies have investigated its characteristics and relationships with food sensory traits. The lack of research on refreshing is perhaps related to the complexity of its perception, as its measurement requires multiple senses to be used simultaneously (Fenko et al., 2009). Refreshing has been considered a holistic descriptor that represents the simultaneous perception of multiple product attributes (Labbe et al., 2009).

### 3.3 Definition of refreshing and associated sensory attributes

**FIGURE 5:**
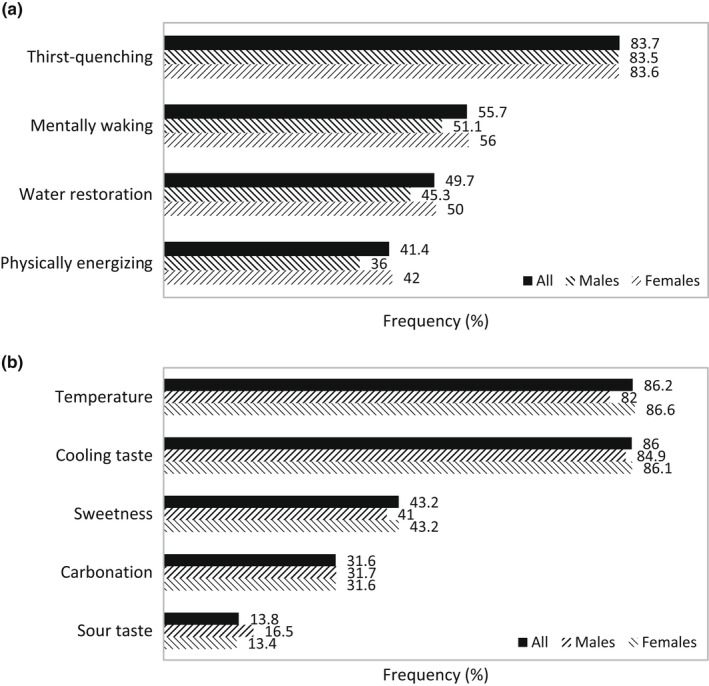
Frequency (%) of terms that define the refreshing perception (a) and factors that drive the refreshing perception (b) according to survey participants (*N* = 1518). *Chi‐square tests were conducted between genders with significance of *p* ≤ .05

Regarding the definition of refreshing, as shown in Figure 5a, thirst‐quenching was noted most frequently (83.7%), followed by mentally waking (55.7%), water restoration (49.7%), and physically energizing (41.4%). The factors that participants felt were important for a food or beverage to be considered refreshing are displayed in Figure 5b. Temperature and cooling taste were considered drivers of refreshing by 86.2% and 86.0% of participants, respectively. Sweetness (43.2%), carbonation (31.6%), and sour taste (13.8%) were selected by the lowest number of participants, indicating that they were not as impactful to the refreshing perception as temperature and cooling taste.

**FIGURE 6:**
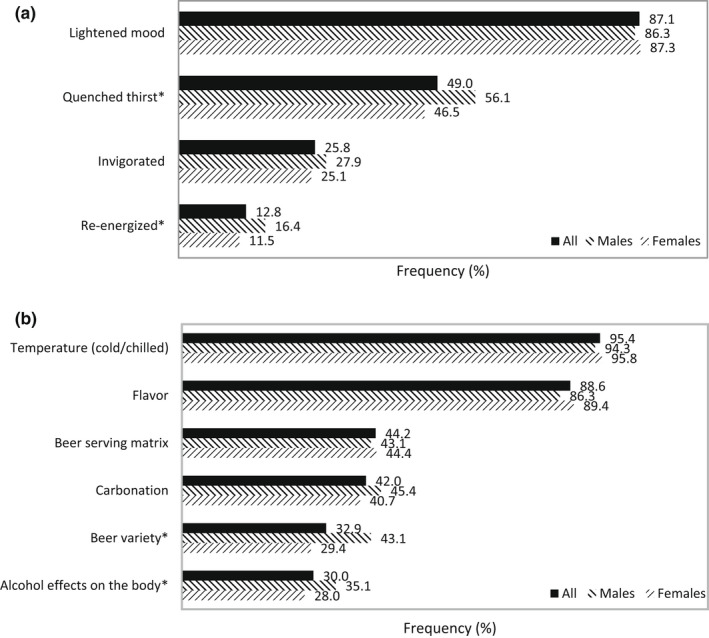
Frequency (%) of terms that define the refreshing perception (a) and factors that drive the refreshing perception (b) according to beer survey participants (*N* = 1050). *Chi‐square tests between genders with significance of *p* ≤ .05

In our beer studies, participants most frequently associated feelings of lightened mood (87.1%) and quenched thirst (49.0%) with the refreshment they perceived from beer (Figure 6a). The top beer properties that had an impact on its refreshment capability were temperature and flavor, according to 95.4% and 88.6% of participants, respectively (Figure 6b).

The concept that refreshing was defined by thirst‐quenching and cooling taste and affected by temperature properties was common among survey participants in our study and was in line with the definitions established by other researchers (De Araujo et al., 2003; Guinard et al., 1998; Labbe et al., 2009; McEwan & Colwill, 1996; van Belzen et al., 2017). Sensory characteristics related to cold temperature were consistently shown to have a positive influence on refreshing (Bouteille et al., 2013; van Belzen et al., 2017), and were associated with refreshment specifically from beer in our survey. Alternatively, thirst, and thereby thirst‐quenching, is usually associated with liquids, which suggests that refreshing is an outcome of fluid intake. This was highlighted by the survey finding that refreshing is an aspect more important in beverages than foods (Figure 2) and underscored by studies that demonstrated high viscosity is a negative driver of refreshing (Guinard et al., 1998; McEwan & Colwill, 1996).

Our survey results pointed to refreshing as being a multisensorial experience involving the tactile perception of temperature and carbonation and the aroma and taste perceptions of flavor (Figure 5). This was in line with the findings that demonstrated refreshing in beers was affected by visual attributes (color and bubble density), olfactory attributes (malty, hoppy, burnt, and metallic), gustatory attributes (acidic and bitter), and mouthfeel attributes (carbonation and viscosity; Guinard et al., 1998). Contribution of flavor to refreshing perception has been reported. Flavored beverages and popsicles could be more refreshing than their nonflavored equivalents (van Belzen et al., 2017). Acidic and mint flavors in particular had positive contributions to the refreshing perception (Fenko et al., 2009; Labbe et al., 2009; McEwan & Colwill, 1996; van Belzen et al., 2017). Flavor influences beer purchasing decisions (Figure 4b) and has a direct impact on whether or not a beer is refreshing (Figure 6b). It has been suggested that acidity in beer was negatively correlated with refreshment, and fruitiness had no correlation (Guinard et al., 1998). This was unexpected even to the researchers, and it should be noted that the beer they used in the study was not representative of the flavor variety available in the market. For that reason, the effect of fruit flavor and acidity on beer refreshment is still unclear.

While it may be related to physiological maintenance such as thirst‐quenching, refreshing has also been linked to emotion, and this emotional viewpoint was supported by survey findings that refreshing is described by mental waking (Figure 5a). Aside from organoleptic sensations, our survey showed that over half of the participants associated refreshment with the experience of cognitive sensations having to do with wakefulness (Figure 5a). Water has been found to be more refreshing when participants were thirsty rather than satiated (De Araujo et al., 2003), which suggests that differing physiological states of the body have distinct effects on refreshing perception. While it is understood that measuring and defining refreshing can lead to ambiguous results, this study found thirst‐quenching, low temperature, and flavor to be prevailing themes that best characterized the refreshing perception.

### 3.4 Refreshing foods and soft drinks

**FIGURE 7:**
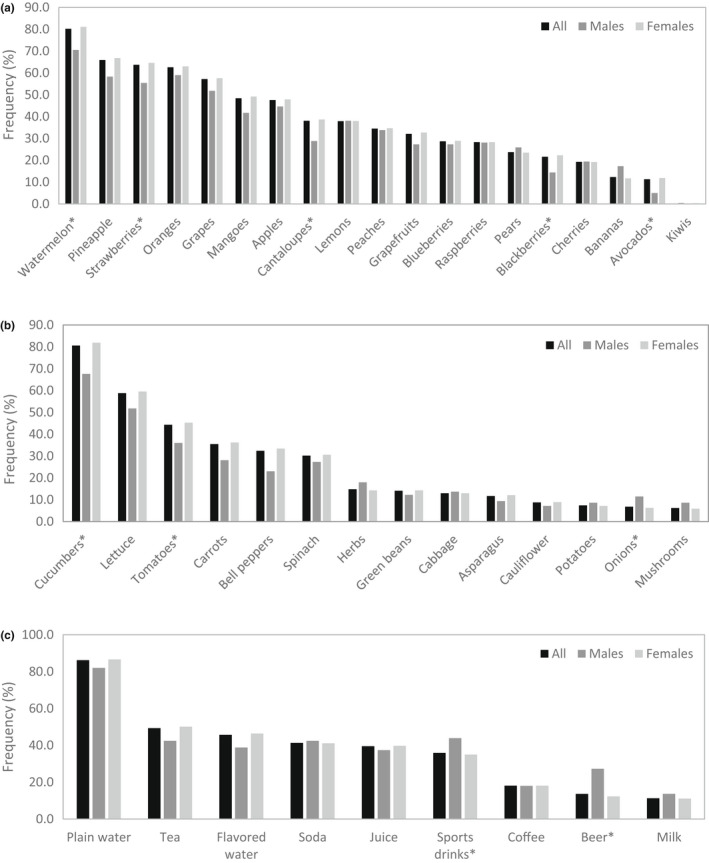
Frequency (%) of fruits (a), vegetables (b), and beverages (c) that are refreshing according to gender (*N* = 1518). *Chi‐square tests were conducted between genders with significance of *p* ≤ .05

As displayed in Figure 7a, watermelon was selected as a refreshing fruit by the greatest number of participants (80.2%), followed by pineapples (65.9%), strawberries (63.7%), oranges (59.0%), and grapes (57.2%). All other fruits were selected by less than half of the participants, with kiwis found refreshing by the lowest number of participants (0.3%). The unusually low selection frequency of kiwis may be connected to its low tendency to be consumed or purchased due to inconvenience and unfamiliarity with the fruit (Baranowski et al., 2008; Harker et al., 2007). For vegetables, cucumbers and lettuce were selected the most (80.6% and 58.8%, respectively), while the others on the list were selected by less than half of the participants (Figure 7b). Cauliflower, potatoes, onions, and mushrooms were selected by less than 10% of the participants (8.8%, 7.4%, 6.8%, and 6.2%, respectively). The beverage most participants felt was refreshing was plain water (86.2%), followed by tea (49.3%), flavored water (45.7%), soda (41.3%), juice (39.5%), sports drinks (35.9%), coffee (18.1%), beer (13.6%), and milk (11.3%), as displayed in Figure 7c.

While the refreshing perception has been linked to flavor factors in beverages and formulated foods (Chaya et al., 2015; Chonpracha et al., 2020; Geier et al., 2016; Mora et al., 2020), its association with fruits is not well understood. This study showed the fruit and vegetable that were most frequently recognized as refreshing were watermelon and cucumber. The classification of watermelon as refreshing has been mentioned by researchers (Liu et al., 2018; Mendoza‐Enano et al., 2019; Tlili et al., 2011) and verified by our sensory studies (Ramirez et al., 2020, 2021). Watermelon and cucumber both belong to the Cucurbitaceae family and have high water contents of ~94% and ~97%, respectively (Olayinka & Etejere, 2018). The data agree in with the survey finding that thirst‐quenching is a defining feature of refreshing. Water content may not be the only determinant of refreshing in fruits, as some with high moisture were not recognized as refreshing. Mushrooms were cited as refreshing by the least number of consumers (6.4%), despite having a water content of ~90% (Zhang et al., 2018). Flavor characteristics likely have an impact on refreshing, as watermelon and cucumber have similar flavor profiles. The major flavor volatiles in watermelon and cucumber are six‐carbon and nine‐carbon volatiles (Beaulieu & Lea, 2006; Fredes et al., 2016), which can contribute melon, green, and fresh notes and could be responsible for the refreshing perception (Ramirez et al., 2020).

Water was considered a refreshing beverage by the greatest number of participants (82.0% and 86.6% males and females, respectively), echoing the results of a survey on refreshing that water was listed by 90% of respondents (Zellner & Durlach, 2002). The universal appreciation for water as a thirst‐quenching beverage is no surprise as it is necessary for body processes and is highly accessible compared to other beverages. Water at 5°C had greater ability to quench thirst compared to water at 22°C (Brunstrom & Macrae, 1997). Water and all beverages in general are likely more refreshing when cold, as implied by the survey finding that temperature and cool taste drive the degree of refreshing (Figure 5b).

### 3.5 Refreshing beers

The first survey showed that only 13.6% participants felt beer was refreshing (Figure 7c), while beer is universally well recognized as a refreshing beverage (Guinard et al., 1998). The reputation of beer refreshing perception was confirmed by the results of the second survey, using participants with regular beer consumption. Of participants in this beer study, 89.8% responded that they consumed beer at least once per month (Figure S1). When questioning was centered on beer in a separate survey, beer was shown to be a major source of refreshment (Figure 4a). The discrepancy could have been due to participants approaching the former question as if they were ranking the choices and determining that water was the superior refreshing beverage.

**FIGURE 8:**
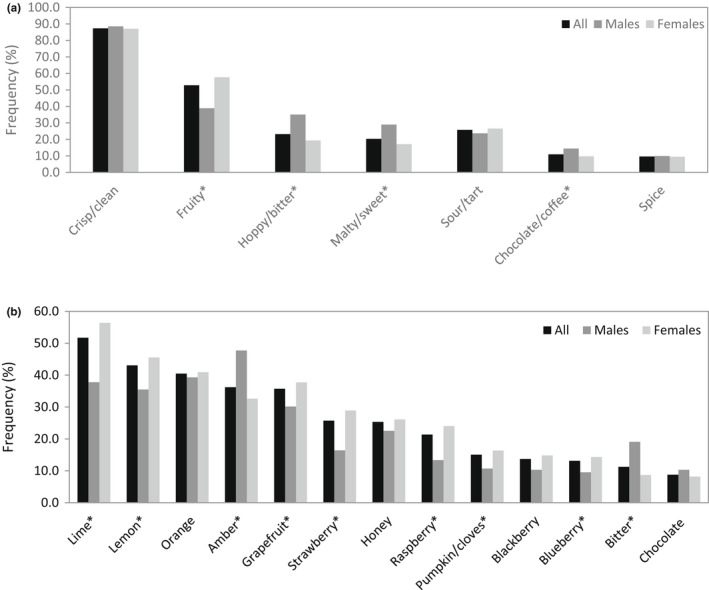
Frequency (%) of flavor profile (a) and flavor type (b) of beers that are refreshing according to gender (*N* = 1050). *Chi‐square tests were conducted between genders with significance of *p* ≤ .05

**FIGURE 9:**
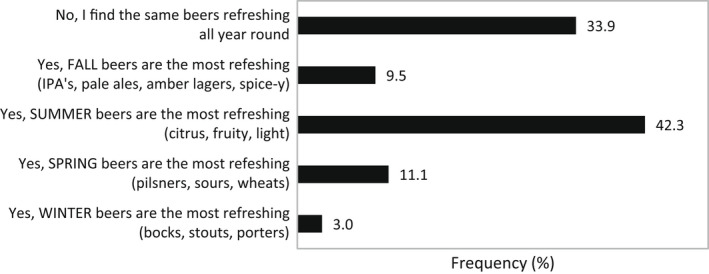
Frequency (%) of participants who perceived season's influence on beer refreshment (*N* = 1050)

**FIGURE 10:**
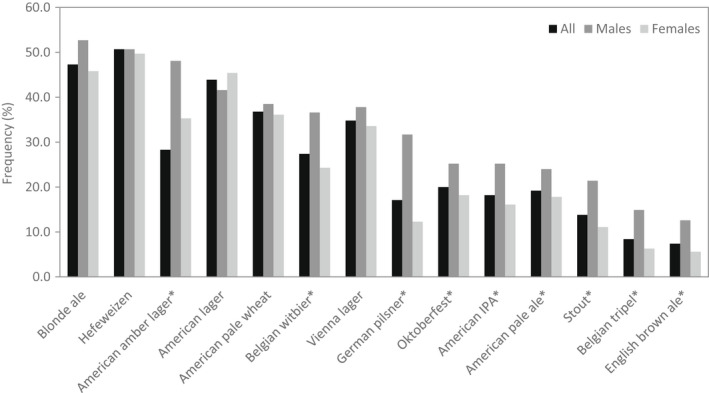
Frequency (%) of refreshing beer varieties according to survey participants (*N* = 1050). *Chi‐square tests were conducted between genders with significance of *p* ≤ .05

When asked which flavor profiles and specific flavors participants found for sensory quality related to refreshing for beer, the majority indicated crisp/clean (87.3%) and fruity (52.9%) flavor profiles and citrus flavors such as lime (51.7%), lemon (43.0%), and orange (40.5%), shown in Figure 8a,b. While more participants noted that summer beers that were fruity and light were more refreshing (42.3%), a close number (33.9%) noted that they found the same beers refreshing, regardless of which season the beer was associated with (Figure 9). As shown in Figure 10, specific beer varieties that were indicated as refreshing by the most participants were Hefeweizen (50.7%), Blonde ale (47.3%), American lager (43.9%), American amber lager (38.3%), American pale wheat (36.8%), and Vienna lager (34.8%).

The beer survey revealed that over half of the participants drink beer to seek a sense of refreshing (Figure 4a). It has been demonstrated that refreshing beers were those with low intensities of overall flavor and malty, burnt, bitter, astringent, acidic, and aftertaste flavors, such as Bud Light and Budweiser (American lagers; Guinard et al., 1998). Survey participants tended to agree with their preferred flavor profile being crisp/clean, rather than beers with spicy, chocolate, coffee notes which were causing strong and heavy perceptions and identified as refreshing by less than 15% of participants (Figure 8a). The varieties most classified as refreshing included Blonde ales and American lagers (Figure 10), which are absent of intense distinct aromas and are generally light in the hoppy, malty, and bitter flavors typical of beer (Guinard et al., 1998). Intensely bitter and hoppy beers such as IPAs (India pale ale) and German pilsners and dark‐colored beers with richer flavors such as stouts, Belgian tripels, and English brown ales were classified as the least refreshing (Figure 10). Sour beers were refreshing according to less than 1% of respondents, suggesting that a dominant acidic profile was a negative driver for refreshing. Coors Brewing Company prides itself in developing “The World's Most Refreshing Beer” and credits the refreshing taste of their Coors Light beer to a light sugar source and pure water source (commercial information), though water itself has no flavor. Along with the survey findings, this suggests that the lack of strong or distinguishing flavor is fundamental for the formulation of a refreshing beer.

Of the flavor profiles other than clean/crisp, a fruity profile stood out as refreshing according to over half of the beer survey participants, with specific fruit flavors being lime, lemon, orange, and grapefruit (Figure 8b). This was in line with the survey finding that participants regard summer beers (citrus, fruity, light) as most refreshing (Figure 9). The affiliation of citrus fruit and the summer season may be attributed to the tropical and subtropical regions where citrus crops are mainly grown. Consumers who live in those hot and humid climates presumably seek thirst‐quenching refreshment by consumption of the pervasive citrus fruits. The ability of acid to provoke saliva stimulation may be connected with the thirst‐quenching and refreshing nature of citrus flavors (Labbe et al., 2009; van Belzen et al., 2017). Oranges, a major citrus, were the fourth most refreshing fruit indicated in the general survey according to 63.1% of participants (Figure 7a). The choice of Hefeweizen beer variety, which was supplemented in the test ballot with the example Blue Moon Belgian White (a beer brewed with orange), was cited as refreshing by the most survey participants (Figure 10). Hefeweizen beers are characterized by intense fruitiness and acidity compared to lagers (Donadini et al., 2013). Orange flavor has been recognized as refreshing in juices, lemon flavor in carbonated drinks and popsicles, and citric acid in edible gels (Fenko et al., 2009; Labbe et al., 2009; McEwan & Colwill, 1996; van Belzen et al., 2017).

### 3.6 Refreshing according to gender

Chi‐square tests did not find significant differences between male and female participants in how they defined the refreshing perception (Figure 5). Thirst‐quenching, temperature, and cooling taste were associated most frequently with the concept of refreshing among both male and female; however, gender was a factor in the refreshing preference for some fruits, vegetables, and beverages (Figure 7). Although appreciation for the refreshing character of watermelon and cucumber was high for both genders, significantly more females chose that fruit and vegetable and chose more fruits and vegetables in general. Watermelon, avocadoes, blackberries, cantaloupes, and strawberries were found to be refreshing by more females than males. Females were more likely to find cucumbers and tomatoes refreshing, while males were more likely to find onions refreshing. This finding is in line with females being more likely than males to consume fruits and vegetables (Baker & Wardle, 2003). Gender correlated with the refreshing preference of beer and sports drinks, which were most selected by males (Figures 7c and 10). Beer was found to be more refreshing by males compared to females, which ties in with greater consumption and preference for beer by males (Klatsky et al., 1990; Ramful & Zhao, 2008). The results suggested that the refreshing perception can vary according to consumer segments.

Gender‐based differences were also found in factors specifically associated with beer consumption reasons (Figure 4) and beer refreshment driving factors (Figure 6). Particular beer variety and locality of beers were more influential on the choice of beers for males compared to females (Figure 4b). A greater number of males than females cited that their reasons for drinking beer were for its taste, refreshing, and alcohol effects on the body. Additionally, more males felt that the refreshing feeling from beer was defined by quenched thirst and replenishment of energy and that the variety of the beer and its alcohol effects had an impact on this perception. The euphoric and relaxing feelings accompanying alcohol consumption had a greater impact on beer refreshment for males compared to females. The impact is likely a positive one, in which stronger alcohol effects on the body relate to a greater refreshing perception, as males find beers with greater alcohol content more appealing (Chrysochou, 2014). Feelings can certainly be unique for each individual, which adds complexity to the refreshing perception. It is similarly noted as regards the subjectivity of the refreshing sensation and the likelihood that its perception is learned (Guinard et al., 1998).

Gender differences were also found to be related to differences in preference of beer flavors (Figure 8). More females indicated that fruity profiles were refreshing, including flavors such as lemon, lime, grapefruit, strawberry, raspberry, blueberry, and pumpkin, while more males preferred a hoppy/bitter, malty/sweet, and deep/chocolate profiles including flavors such as bitter and amber. The observation that more females consider fruity beers refreshing compared to males may be associated with fruit intake being greater for females and females preferring sweeter beers (Baker & Wardle, 2003; Muggah & McSweeney, 2017). Significantly more males considered a hoppy/bitter profile and stronger, more flavorful beers to be refreshing compared to females, which may be tied to gender differences in bitter perception, with males being less likely to sense bitterness and, therefore, more likely to accept bitter beers (Bartoshuk et al., 1994).

Refreshing preference for majority of beer varieties was dependent on gender. A greater number of males selected varieties of Hefeweizen (50.7%), Blonde ale (47.3%), American lager (43.9%), American amber lager (38.3%), American pale wheat (36.8%), and Vienna lager (34.8%). American lagers, Blonde ales, American pale wheat, Vienna lagers, and Hefeweizens were equally preferred by both genders (Figure 10). Few studies have investigated the influence of gender on consumer choices of beer varieties. The different preferences of beer varieties between males and females might be due to the flavor of beer, as aforementioned gender preference for beer flavor (Figure 8). Gender‐based differences in taste, smell, trigeminal, and oral‐somatosensory perception have been reported (Betancur et al., 2020), consequently leading to different preferences or choices of food.

### 3.7 Refreshing according to age

**FIGURE 11:**
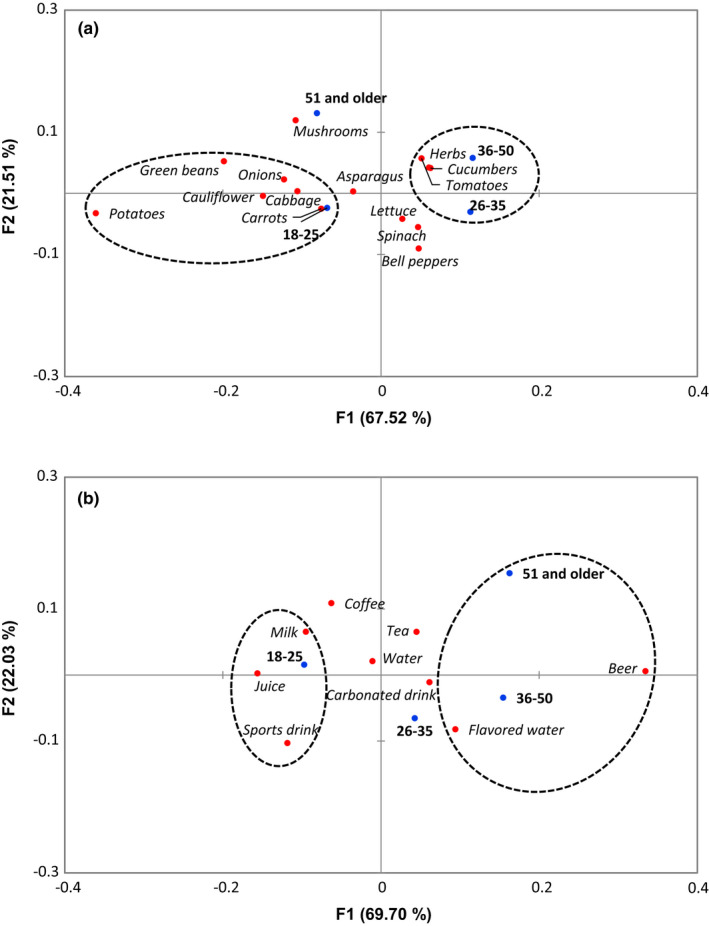
The first two dimensions of correspondence analysis (CA) symmetric plot using age as Rows and vegetables (*p* = .005) (a) and soft drinks (*p* < .0001) (b) as Columns (*N* = 1518). Oval circles were created based on XLSTAT CA output of “Squared cosines of the Rows” and “Squared cosines of the Columns” which showed significant separation at the first two components

Correspondence analysis allowed the visualization of survey responses according to the participant age groups. According to the analysis, the refreshing perception, including definition of refreshing and factors that cause refreshing, was not significantly different among the age groups (*p* > .05, Figure S2). Particular preferences for refreshing fruits, vegetables, and beverages were found to be significantly different among some age groups (*p* < .05). Participants in the 18‐ to 35‐year‐old age group were more likely to consider carrots, lettuce, spinach, and bell peppers to be refreshing compared to the other age groups, while those 36 and older were more likely to consider mushrooms and herbs to be refreshing (Figure 11a). Those in the 18‐ to 25‐year‐old group were more likely to consider juice to be refreshing; those in the 26‐ to 35‐year‐old group considered sports drinks and flavored water to be refreshing; those in the 36‐ to 50‐year‐old group considered carbonated drinks and beer to be refreshing; and those 51 or older considered coffee and tea to be refreshing (Figure 11b).

**FIGURE 12:**
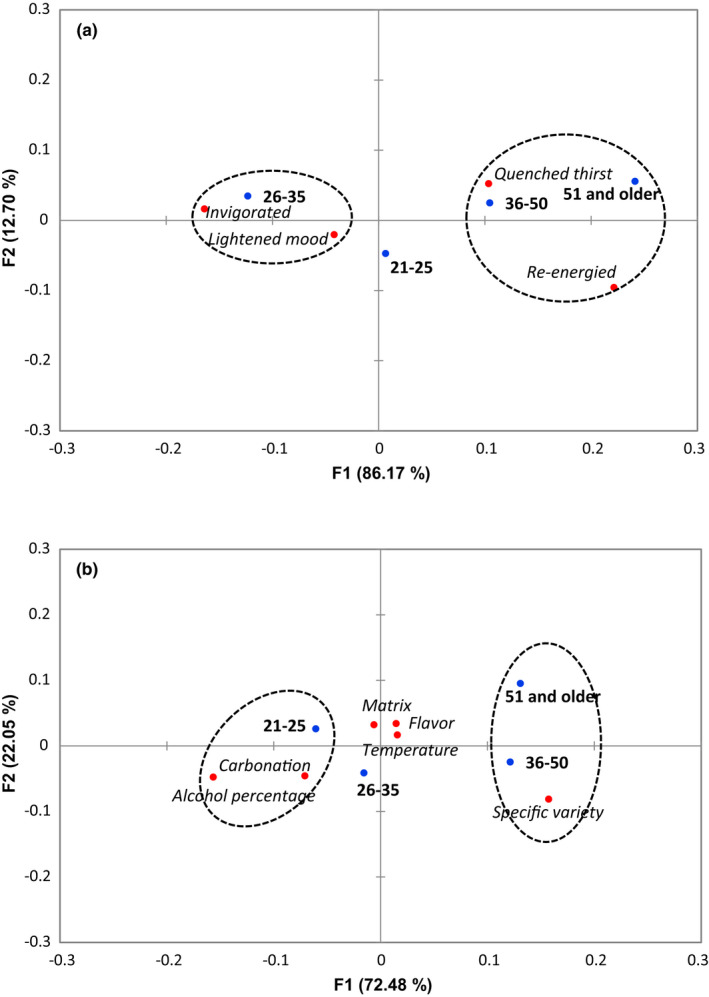
The first two dimensions of correspondence analysis (CA) symmetric plot using age as Rows and refreshing perception (*p* = .004) (a) and refreshing factor (*p* = .037) (b) as Columns (*N* = 1050). Oval circles were created based on XLSTAT CA output of “Squared cosines of the Rows” and “Squared cosines of the Columns” which showed significant separation at the first two components

**FIGURE 13:**
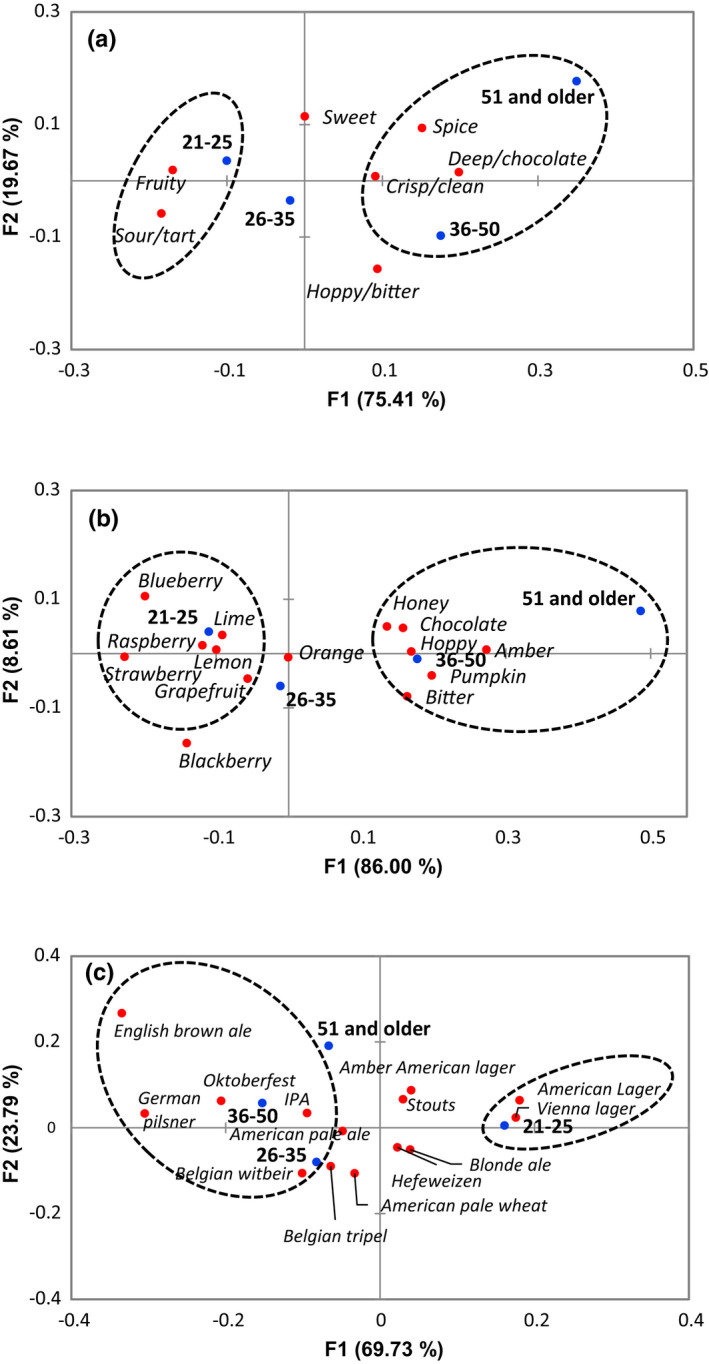
The first two dimensions of correspondence analysis (CA) symmetric plot using age as Rows and beer flavor profile (*p* < .0001) (a), beer flavor (*p* < .0001) (b), and beer variety (*p* < .0001) (c) as Columns (*N* = 1050). Oval circles were created based on XLSTAT CA output of “Squared cosines of the Rows” and “Squared cosines of the Columns” which showed significant separation at the first two components

Responses to all questions from the beer survey were found to differ significantly based on age group (*p* < .05). For the feelings and factors associated with the refreshing perception of beer, participants in the 21‐ to 25‐year‐old group were more likely to associate lightened mood with the refreshing perception from beer and noted that the serving glass, flavor, and temperature of the beer had an impact (Figure 12a,b). Participants in the 26‐ to 35‐year‐old group were more likely to associate invigoration and noted that carbonation and alcohol percentage influenced refreshing. Those 36 or older were more likely to associate quenched thirst and found specific variety of the beer to be important to the refreshing perception. Participants in the 21‐ to 35‐year‐old groups were more likely to regard fruity and sour/tart flavor profiles as refreshing compared to those in the 36 or older groups, who were more likely to regard hoppy/bitter, sweet, and spice profiles as refreshing (Figure 13a). Those results were reflected in the responses related to specific beer flavors, which showed that 21‐ to 35‐year olds were more likely to consider lime, lemon, raspberry, and grapefruit as refreshing, while participants in the 36 or older groups considered hoppy, amber, chocolate, and pumpkin as refreshing (Figure 13b). There was a clear difference in refreshing perception of different beer varieties according to age group (Figure 13c). Participants 21–25 years of age were more likely to indicate that American and Vienna lagers were refreshing, 26‐ to 35‐year olds indicated Belgian tripel, Belgian witbeir, and American pale wheat, 36‐ to 50‐year olds indicated IPA and German pilsner, and 51 and older indicated English brown ale were refreshing.

Age has been shown to be a significant variable in food choice decisions (Chambers et al., 2008). The effects of aging can be at the biological level (a gradual decline in the sensitivity of taste and smelling) and the behavioral factor such as health consideration in older population (Chambers et al., 2008). This may explain shifts in preferences as consumers age; however, food choice throughout the life span would also depend on other demographic variables such as knowledge and personal economic status (Betancur et al., 2020). In this study, four age groups showed a significantly different opinion toward refreshing perception of different foods and their choice was most likely influenced by their familiarity or consumption frequency of the products.

Knowledge about age influencing beverage choice is less than age difference for food choice (Mueller Loose & Jaeger, 2012), and there are few studies specifying the influence of age variable on consumer choice of beer (Betancur et al., 2020). The decreased sensory perception and appreciation of drinks in the elderly could cause a change in the patterns of beer consumption in quantity and variety. Age causes a general linear decline of beer consumption along with increasing age (Kerr et al., 2004), while aging also results in a shift toward beer with stronger tastes/flavors, which are appreciated with diminished sensory ability (Betancur et al., 2020). These recorded literature are consistent with the findings from current study that four age groups had different preference toward refreshing beers.

## 4 CONCLUSIONS

Surveys investigated consumer perspective of refreshing perception in general and specifically in beers. The main themes of consumer definitions of refreshing agreed with definitions of other researchers: thirst‐quenching/water was refreshing, cold was more refreshing than warm, flavor had an impact. There were no gender differences in thirst‐quenching definitions and cold association with refreshing; however, there were gender differences in the types of foods and flavors that impact refreshing perception.

Findings from this study contribute to the understanding of the underexplored concept of refreshing, an emotion‐associated attribute of food and drinks. Manufacturers can apply this research for informed development of refreshing beverages and beers for specific functions such as improving cognitive performance. The findings of varying consumer segments in age and gender preference on refreshing‐related food, beverage, and beer could be used to design the personalized food products to target a specific market.

The potential limit of the current study included bias from gender, though the statistical test power for male participant was high. Additionally, this study used self‐report measures (e.g., sensory, consumer test, focus group, questionnaires), which are the most common methods used to evaluate emotions evoked by food experiences, due to their ease of application, cost‐effectiveness, and discriminative power. In the future study, it would be significant to incorporate different approaches across multiple disciplines, such as physiological system response and brain imaging techniques, which can directly access people's primary response to an emotional stimulus without involving a conscious process.

## CONFLICT OF INTEREST

No conflicts of interest to declare.

## Supporting information

Figure S1‐S2

## Data Availability

Our data are available for review.
