# Supplementary material for: Examining the consumer view of refreshing perception, relevant fruits, vegetables, soft drinks, and beers, and consumer age and gender segmentations
Source: Food Sci Nutr. 2022 Mar 29;10(8):2516–31. doi: 10.1002/fsn3.2857 (PMC9361463; doi:10.1002/fsn3.2857)
Supplement: Supplementary file 1 — Figure S1‐S2 [file FSN3-10-2516-s001.docx]

**Supplemental Documents:**

**Figure S1**. Consumer beer consumption frequency (N=1050).

**Figure S2.** The first two dimensions of correspondence analysis (CA) symmetric plot using age as Rows and a combination of definition and impact factors as Columns (*p*=0.087, N=1518).
